# Dataset on Trombe wall application in a factory building

**DOI:** 10.1016/j.dib.2024.110196

**Published:** 2024-02-17

**Authors:** Aleksejs Prozuments, Guna Bebre, Mohamed Tariq Ekeramodien Kahn

**Affiliations:** a Department of Heat Engineering and Technology, Faculty of Civil Engineering, Riga Technical University, Riga LV-1048, Latvia; b Department of Electrical, Electronic and Computer Engineering, Faculty of Engineering and the Built Environment, Cape Peninsula University of Technology, Cape Town 7535, South Africa

**Keywords:** Solar energy, Solar thermal, Space heating, Passive solar energy utilization

## Abstract

An experimental passive solar thermal transmission wall (also known as Trombe wall) prototype was installed on a factory building wall in Kalnciems, Latvia to carry out temperature measurements at different representative locations in order to evaluate Trombe wall's potential to be used as a secondary space heating source. The average winter temperature in Kalnciems is 0.7 °C which stipulates the need for space heating (6-7 months a year). The dataset collection spanned from March 2022 through July 2023, enabling to acquire data over meaningful timeframe, as well as one full year cycle. Exploring Trombe wall technology in a cold climate setting aimed to examine its potential to curtail space heating energy consumption. The experimental Trombe wall prototype was closely monitored, and showcased substantial temperature increase during sunlight hours, emphasizing its potential to be used as a secondary heating source in industrial settings. The Trombe wall structure also demonstrated a sufficient internal temperature gradient (Δt between 1.5 and 5.0 m level a.g.) and generated rather substantial energy output, which is especially important for regions with moderate and cold climates. Enlarging and refining these systems could significantly reduce costs associated to space heating in industrial settings. Recommendations include investigating enhanced air circulation and filtration for improved functionality and user comfort.

Specifications Table

**Table:** 

Subject	Renewable Energy, Sustainability and the Environment.
Specific subject area	Evaluating Trombe wall application and potential as secondary space heating source in moderate/cold climate region
Data format	Raw data (csv.)
Type of data	Tables
Data collection	The following continuous temperature measurements were carried out at 10-min intervals throughout March 2022 and July 2023: ■ outdoor temperature; ■ indoor temperature (near the working area); ■ temperature inside the Trombe wall structure at 1.5 m height; ■ temperature inside the Trombe wall structure at 5 m height; ■ supply air temperature (after the duct fan). The incident solar radiation (W/m^2^) onto the Trombe wall structure was also monitored.Instruments used: ■ Temperature: HOBO MX1101 Temp/RH Data Logger; ■ Solar Radiation: S-LIB-M003 (Silicon Pyranometer) Smart Sensor
Data source location	Kalnciems, Latvia (56.79600001667912, 23.618391082186534)
Data accessibility	Data identification number: https://data.mendeley.com/datasets/56m6bv8c7w/1
Related research article	Prozuments, A.; Borodinecs, A.; Bajare, D. Trombe Wall System's Thermal Energy Output Analysis at a Factory Building. Energies 2023, 16, 1887. https://doi.org/10.3390/en16041887

## 1 Value of the Data

• The dataset provides comprehensive information on the performance of Trombe wall technology in a cold climate setting, offering valuable insights into its potential as a supplementary space heating system. By demonstrating the performance of the experimental Trombe wall in maintaining indoor temperatures and potentially reducing heating energy consumption, the dataset contributes to enhanced understanding of sustainable heating solutions, particularly in regions with challenging climatic conditions.
• The dataset offers real-world temperature measurements for refining Trombe wall designs. Researchers can utilize this dataset to analyze temperature variations and calculate energy yields in industrial (as well as residential, commercial) settings across various climate regions. On top of that data serves as a valuable resource for refining Trombe wall designs, optimizing their performance, and enhancing their effectiveness in different environmental contexts. By studying the temperature gradients and energy outputs measured in this study, researchers can develop improved Trombe wall configurations tailored to specific climate conditions, thereby advancing the adoption of sustainable heating technologies in diverse settings.
• The dataset serves as a benchmark for future research on passive heating solutions, providing a reference point for evaluating the performance of Trombe walls and comparing them with other alternative heating systems. Researchers can use this dataset to assess the efficacy of Trombe walls in different climatic conditions, explore modifications to Trombe wall structures, and investigate innovative approaches to passive heating. By building upon the findings of this study, future research can further advance our knowledge of passive heating technologies and contribute to the development of more sustainable building practices.
• The dataset offers empirical evidence to support assessments of the cost-effectiveness of Trombe wall integration in factory environments. By analyzing the energy savings and thermal performance data provided in this study, researchers can conduct economic viability assessments for similar projects and evaluate the long-term benefits of adopting Trombe wall technology. These data enable stakeholders to make informed decisions regarding the implementation of Trombe walls as a sustainable heating solution, considering both their environmental impact and economic feasibility.
• Finally, the dataset has the potential to inform broader research efforts on sustainable heating strategies and renewable energy utilization. By highlighting the effectiveness of Trombe walls in reducing heating energy consumption and minimizing environmental impact, this dataset contributes to ongoing discussions about the transition to renewable energy sources and the development of more sustainable building practices. Researchers, policymakers, and industry professionals can use this dataset to explore innovative approaches to heating and cooling, ultimately advancing efforts to mitigate climate change and promote environmental sustainability.

## 2 Background

As the cost of fossil fuel-based energy sources is continuously rising, passive carbon-free energy generation and storage is becoming an increasingly appealing and feasible concept . One of such concepts is a passive solar thermal transmission wall known as Trombe wall technology. It is a passive solar thermal energy storage unit that is utilized to offset building heating loads in an innovative and environmentally friendly way in order to reduce building energy consumption (electricity, gas, etc.) for space heating. Depending on the external climate and the desired level of indoor comfort, the Trombe wall may be combined with an alternative heating system. Consequently, the Trombe wall is typically used as a supplementary system in medium-temperature and cold regions to save building heating energy during the cold period of the year.

## 3 Data Description

**Table:** 

Trombe Wall—Real-Operating Setup	
Location	Kalnciems, Latvia
Position	Fully vertical
Orientation	Due east
Operation	Passive solar heat transmission; fan-enforced air circulation ∼200 m^3^/h
Position towards outdoor weather conditions/elements	Fully exposed
Height, mm	5400
Width, mm	4000
Surface area, m^2^	21.6
Depth, mm	70 (50 + 40/0)
Volume of air gap, m^3^	0.69
Measurement, unit	Temperature,°C
No. of measurement points	4
Backwall material	Sheet metal plates
Glazing material	Polycarbonate sheets
Frame material	Timber
Backwall material	Corrugated, galvanized and painted sheet metal
Measurement timeframe	Mar 2022—Jul 2023

## 4 Experimental Design, Materials and Methods

The Trombe wall system was integrated into a 1000 m^2^ factory building in Kalnciems, Latvia, converted from a sheet metal hangar to a timber-frame modular house component production facility. The factory features poor air-tightness and lack of thermal insulation, leading to indoor temperatures being heavily influenced by outdoor conditions, oftentimes falling below workplace temperature thresholds in colder season (typically lasting from mid-October until early April), stipulating the need for space heating.

The Trombe wall prototype was installed on a fully vertical façade of the factory building, oriented eastwards, and supplemented by fan-enforced air circulation (+/-200 m^3^/h). The wall's height - 5400 mm, width - 4000 mm, with a total surface area of 21.6 m^2^ and an air gap volume of 0.69 m^3^. Temperature measurements were conducted at various points: indoor and outdoor temperature, along with temperatures at different heights within the Trombe wall structure (1.5 m and 5 m), as well as supply air temperature (directly after fan). A vent and supply duct fan, installed at 5 m above the ground, facilitated the discharge of accumulated thermal air mass into the factory, specifically to the work zone. A 100 mm air channel within the Trombe wall housed the duct fan, directing heated air to the factor's working area through an insulated duct.

Continuous temperature measurements, taken at 10 min intervals, encompassed outdoor and indoor temperatures, temperatures within the Trombe wall at 1.5 m and 5 m heights, and supply air temperature post-fan. Incident solar radiation (W/m^2^) onto the Trombe wall structure was also monitored.

Experimental setup included detailed specifications of materials (timber framing, polycarbonate glazing, dark corrugated metal sheet backwall), sensors' positioning, and airflow control for comprehensive data collection.

## Limitations

1. Different dataset measurements were carried out at different timeframes due to limited sensor availability and technical constraints due to factory operation specifics (until 5/5/22), with solar radiation monitor only being available starting from 27/07

**Figure:**
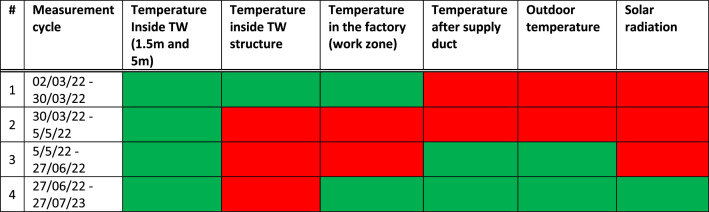

2. The factory wall upon which the Trombe wall prototype was installed is facing East, resulting in the reduction of the potential generated and thus transferred solar heat gain.

## Ethics Statement

**T**he authors have read and follow the ethical requirements for publication in Data in Brief and confirming that the current work does not involve human subjects, animal experiments, or any data collected from social media platforms.

## CRediT authorship contribution statement

**Aleksejs Prozuments:** Conceptualization, Methodology, Writing – original draft. **Guna Bebre:** Data curation, Visualization. **Mohamed Tariq Ekeramodien Kahn:** Writing – review & editing.

## Data Availability

Dataset on Trombe Wall Application in a Factory Building (Original data) (Mendeley Data)

## Appendix Supplementary materials

Image, application 1

